# Studying the microbiota of bats: Accuracy of direct and indirect samplings

**DOI:** 10.1002/ece3.4842

**Published:** 2019-01-24

**Authors:** Muriel Dietrich, Wanda Markotter

**Affiliations:** ^1^ Department of Medical Virology, Faculty of Health Sciences, Centre for Viral Zoonoses University of Pretoria Pretoria South Africa; ^2^ UMR PIMIT (Processus Infectieux en Milieu Insulaire Tropical), INSERM U1187, CNRS UMR 9192, IRD UMR 249 Université de la Réunion Sainte‐Clotilde Reunion Island France

**Keywords:** 16S rRNA sequencing, bats, feces, Illumina, South Africa, urine

## Abstract

Given the recurrent bat‐associated disease outbreaks in humans and recent advances in metagenomics sequencing, the microbiota of bats is increasingly being studied. However, obtaining biological samples directly from wild individuals may represent a challenge, and thus, indirect passive sampling (without capturing bats) is sometimes used as an alternative. Currently, it is not known whether the bacterial community assessed using this approach provides an accurate representation of the bat microbiota. This study was designed to compare the use of direct sampling (based on bat capture and handling) and indirect sampling (collection of bat's excretions under bat colonies) in assessing bacterial communities in bats. Using high‐throughput 16S rRNA sequencing of urine and feces samples from *Rousettus aegyptiacus*, a cave‐dwelling fruit bat species, we found evidence of niche specialization among different excreta samples, independent of the sampling approach. However, sampling approach influenced both the alpha‐ and beta‐diversity of urinary and fecal microbiotas. In particular, increased alpha‐diversity and more overlapping composition between urine and feces samples was seen when direct sampling was used, suggesting that cross‐contamination may occur when collecting samples directly from bats in hand. In contrast, results from indirect sampling in the cave may be biased by environmental contamination. Our methodological comparison suggested some influence of the sampling approach on the bat‐associated microbiota, but both approaches were able to capture differences among excreta samples. Assessment of these techniques opens an avenue to use more indirect sampling, in order to explore microbial community dynamics in bats.

## 1 INTRODUCTION

Bats are geographically widespread and play an important role in many ecosystems (Boyles, Cryan, McCracken, & Kunz, 2011; Hodgkison, Balding, Zubaid, & Kunz, 2003), but relatively little is known about the ecology of their microbial communities and the role these could play in bat health and behavior. The recent bat‐associated disease outbreaks in humans (e.g., SARS, MERS, Marburg) have however stimulated research on microbial community dynamics in bats (Calisher, Childs, Field, Holmes, & Schountz, 2006), but analyses of their microbiotas, based on high‐throughput sequencing technology, remains rare. In particular, more research is needed on the microbiota residing in bat's excreta such as urine and feces, which are potentially involved in pathogen transmission (Dietrich, Kearney, Seamark, Paweska, & Markotter, 2018).

Our ability to characterize the microbiota of bats can be restricted by the challenge of collecting samples in the field, especially when non‐lethal methods are prioritized. Some studies, investigating the bacterial microbiota of bats, have focused on digestive tract‐derived samples and have used the capture and euthanasia of individuals (Banskar, Mourya, & Shouche, 2016; Carrillo‐Araujo et al., 2015; Phillips et al., 2012). In contrast, other studies were based on the capture and release of bats, using urine and fecal samples directly collected in hand from the animal (Dietrich, Kearney, Seamark, & Markotter, 2017; Dietrich et al., 2018; Hughes, Leech, Puechmaille, Lopez, & Teeling, 2018; Veikkolainen, Vesterinen, Lilley, & Pulliainen, 2014; Uddin et al., 2018). In the field, such direct sampling is not always effortless, as not all bat species urinate and defecate easily when captured. Therefore, alternative indirect approaches have been used to specifically study bacterial communities in bat guano, either through the collection of fresh guano pellets using collection plates (Banskar, Bhute, Suryavanshi, Punekar, & Shouche, 2016; Henry et al., 2018), or the sampling of the cave floor (De Mandal, Panda, Bisht, & Kumar, 2015). The indirect collection of bat feces, but also urine droplets, has already shown to be a good proxy to study the temporal variation of bacterial and viral prevalence within bat colonies (Dietrich et al., 2015; Drexler et al., 2011). There is now a need for assessing the accuracy of such indirect sampling to investigate the bacterial microbiota of bats based on high‐throughput sequencing tools. This will allow to test whether indirect sampling can be implemented in further investigations of microbiota in bat populations.

In this study, we analyzed a South African population of the well‐known reservoir of Marburg virus, the cave‐dwelling fruit bat species, *Rousettus aegyptiacus*. We compared the use of direct (from bat handling) and indirect (requiring no capture) sampling in bats to investigate bacterial communities using high‐throughput sequencing technology. The main objective was to determine whether indirect sampling would produce similar results to direct sampling. We first assessed the accuracy of both type of approaches to evaluate niche specialization of the microbiota, based on urine and feces individual samples. We then looked at differences in microbiota diversity and composition and attempted to identify potential biases in both approaches.

## 2 MATERIALS AND METHODS

### 2.1 Sample collection

Bat sampling was conducted in a maternity colony of *Rousettus aegyptiacus*, located in Matlapitsi Cave, Limpopo province (S 24.11483; E 30.12110), South Africa, on two occasions: January 2016 and April 2016. For direct sampling, bats were captured using harp‐traps placed at the entrance of the cave and each individual bat was placed directly in a clean numbered non‐sterile cotton cloth bag, that was not re‐used to prevent cross‐contamination between bat individuals. Bats were immediately processed on site under biosafety conditions, including Tyvek suits coupled with powered air‐purifying respirators. We collected urine and feces while handling bats. When a urine droplet was available, it was collected using a pipet at the urethral opening. Feces droppings, when available, were collected using sterilized clean forceps. All bats were released after sampling.

For indirect sampling, we used four plastic film‐covered cardboard rectangles (100 × 50 cm) placed inside larger plastic trays. Before use, the surface was wiped with a DNA decontaminant reagent (Sigma‐Aldrich). Trays were positioned under the bat colony in the cave during the day (following the night of direct sampling), and left for 20 min. This time point was chosen to obtain enough samples, while minimizing the disturbance of the bat colony. Urine droplets were collected using a pipet. However, because feces droppings were not present in large numbers on the trays, we sampled droppings directly on the cave floor with sterile swabs (Swabs 150C, Copan). Fresh droppings were selected based on their visual aspect (material not dried). We carefully sampled only the top of the droppings, to prevent the contact of the swab with floor material. Each sample was placed in a sterile vial (Sarstedt) and stored in liquid nitrogen prior to the transfer to a −80°C freezer.

The sampling protocol was approved by the University of Pretoria Animal Ethics committee (EC054‐14) following guidelines of the South African National Standard (SANS 10386:2008). Catching and collecting was carried out in strict accordance with the terms of the research permit CPB6‐003767 issued by the Department of Economic Development, Environment & Tourism (Limpopo province) and the Department of Agriculture, Forestry and Fishery section 20 approval.

### 2.2 Sample processing

DNA extraction was performed as detailed previously (Dietrich et al., 2017), using a ZR‐Duet DNA/RNA MiniPrep Plus kit (Zymo Research), and a pre‐treatment for robust lysis of Gram‐positive and Gram‐negative bacteria. We included three negative controls during DNA extraction to control for the presence of exogenous DNA in laboratory reagents and materials. Eluted DNA was then used for Illumina sequencing of 16S amplicons in a single run, including a negative PCR control to further identify potential exogenous bacterial DNA introduced during library preparation, as described previously (Dietrich et al., 2017).

### 2.3 Bioinformatic and statistical analyses

Bioinformatic and statistical analyses were performed as described previously (Dietrich et al., 2017), with MOTHUR v.1.38.1 following the MiSeq SOP Pipeline (Kozich, Westcott, Baxter, Highlander, & Schloss, 2013; Schloss et al., 2009) and R software (R Core Team, 2013), primarily with the vegan, Rcmdr and ggplot2 packages (Fox, 2005; Oksanen et al., 2015; Roberts, 2015; Wickam, 2016). Assembled reads were quality trimmed based on their length prior to alignment against the MOTHUR‐formatted SILVA database. Preclustering of the data was performed using a 4‐bp difference, following by the detection and removal of chimeras using the UCHIME algorithm (Edgar, Haas, Clemente, Quince, & Knight, 2011). We then classified sequences using the MOTHUR‐formatted version of the RDP training set (v.9), and any unknown, chloroplast, mitochondrial, archaeal, or eukaryotic sequences were removed. Sequences were clustered into phylotypes using a 97% identity threshold. Based on the analysis of negative controls, potential exogenous phylotypes were identified using the same approach as in Dietrich et al. (2017), and we produced four datasets corresponding to different levels of exogenous DNA removal (i.e., none, two, 51 and all exogenous phylotypes removed). After checking for consistency among results from these four datasets, results are presented for the dataset where exogenous phylotypes with a relative abundance >10% in the controls (*n* = 2) were removed. Phylotype tables were rarefied at the smallest library (8,340 sequences per sample when urine and feces samples were analyzed together, 8,340 and 18,727 sequences per sample for the separate analyze of urine and feces datasets, respectively).

We calculated alpha‐diversity using the inverse Simpson diversity index and performed comparison between excreta samples (i.e.*,* urine and feces) using a Generalized Linear Model (GLM) including a Gaussian distribution and sampling month as an explanatory variable (Supporting Information Table S1, available from the Dryad Digital Repository: https://doi:10.5061/dryad.k2v2006). To analyze the structure of microbiota, we used beta‐diversity measurements and tested structure among the two excreta samples. For that, permutational MANOVA (PERMANOVA) tests with 999 permutations were performed and non‐metric multidimensional scaling (NMDS) ordinations were conducted on Bray–Curtis dissimilarities, calculated from rarefied sequence counts, after square root transformation and Wisconsin standardization, to produce plots.

Then, we tested difference of alpha‐diversity between sampling approaches (direct vs. indirect), for urine and feces samples separately, using the same procedure of GLM as above (Supporting Information Table S1). The influence of sampling approach on beta‐diversity was then tested using PERMANOVAs and plotted with NMDS. We used linear discriminant analysis effect size (LEfSe; Galaxy v.1.0) to identify phylotypes that differed significantly in relative abundance between sampling approaches (Afgan et al., 2018; Segata et al., 2011). We set the alpha value for the Kruskal–Wallis test at 0.05 and the threshold on the logarithmic LDA score at 2.0. We calculated the proportion of shared phylotypes between urine and feces samples for each sampling approach, and compared it using a fisher test.

## 3 RESULTS

### 3.1 Bat samples and phylotype classification

A total of 35 bat samples were collected, including 21 samples (16 urine and five feces) directly from bats in hands, and 14 other samples (eight urine and six feces) using indirect sampling in the cave (Supporting Information Table S2, available from the Dryad Digital Repository: https://doi:10.5061/dryad.k2v2006). The average number of assembled reads per sample was 31,950 and was not different between sampling approaches (ANOVA: *F* = 0.445, *p* = 0.509). Bacterial communities in the 35 bat samples and the 3 DNA extraction negative controls were classified into 748 phylotypes. Among them, 24% (*n* = 177) were present in the negative controls, with the genera *Escherichia‐Shigella* and *Pseudomonas* being the two most dominant phylotypes. Further results are presented when both these phylotypes were removed from the dataset.

### 3.2 Assessment of niche specialization

**Figure 1:**
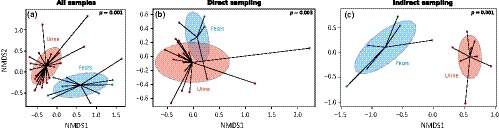
Niche specialization of microbiota when (a) all samples were included, or either with (b) direct or (c) indirect sampling approach. Each point represents a sample and shaded ellipses represent one standard deviation around sample group centroids. The *p*‐value of the PERMANOVA is indicated on the right corner

When samples were analyzed altogether, we found evidence of niche specialization (PERMANOVA: *R* ^2^ = 18%, *p* = 0.001, Figure 1a), with the highest level of alpha‐diversity found in urine compared to feces samples (GLM_1_: $\chi_{1}^{2}$ = 631.26, *p* = 0.021; Supporting Information Table S1). When analyzed separately, both sampling approaches performed well in assessing niche specialization, but it was more evident with indirect (*R* ^2^ = 39%, *p* = 0.001, Figure 1c) than direct (*R* ^2^ = 15%, *p* = 0.003, Figure 1b) sampling.

### 3.3 Influence of the sampling approach

**Figure 2:**
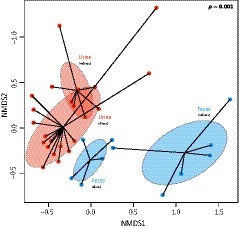
Microbiota structure according to excreta sample (urine vs. feces) and sampling approach (direct vs. indirect). Each point represents a sample and shaded ellipses represent one standard deviation around sample group centroids. The *p*‐value of the PERMANOVA is indicated on the right corner

**Figure 3:**
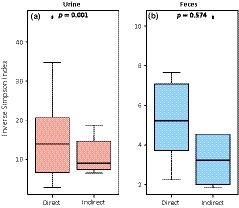
Difference of microbiota diversity between direct and indirect samplings, for (a) urine and (b) feces. The *p*‐value of the GLM is indicated at the top of the box

Sampling approach was a major determinant in structuring the microbiota (*R* ^2^ = 31%, *p* = 0.001, Figure 2). Given the difference of microbiota among excreta samples, the influence of sampling approaches (direct vs. indirect) was then tested for each excreta separately. For urine, although some overlap was observed in the NMDS plot (Figure 2: in red), sampling approach significantly influenced the urinary microbiota (*R^2^* = 16%, *p* = 0.001), for which alpha‐diversity was higher when samples were collected directly (GLM_2_: $\chi_{2}^{2}$ = 829.41, *p* = 0.001, Figure 3a and Supporting Information Table S1). In contrast, the NMDS plot for feces showed very distinct microbiota between sampling approaches (Figure 2: in green), but the difference was not statistically significant (*R* ^2^ = 14%, *p* = 0.154). Moreover, even if alpha‐diversity tend to be also higher in feces directly collected from bats (Figure 3b), this was not statistically significant (GLM_3_: $\chi_{2}^{2}$ = 2.578, *p* = 0.574, Supporting Information Table S1). Finally, when direct sampling was used, slightly more phylotypes (51%, *p* = 0.010) were shared between urine and feces samples, compared to indirect sampling (48%).

LEfSe analysis of urine samples identified 103 phylotypes that were differentially distributed between sampling approaches (Supporting Information Figure S1). For example, urine samples collected directly from bats were enriched in *Streptococcus*, while bacteria such as *Gracilimonas* and *Salinisphera* were more abundant in urine collected in the cave. It is important to note that *Streptococcus* was the most abundant phylotype in feces, for both direct (39.4%) and indirect (44.5%) samplings. In feces, LEfSe analysis revealed 88 phylotypes significantly enriched when using direct sampling (mainly *Actinobacillus* and members of Pasteurellaceae), and only five phylotypes (such as *Nesterenkonia *and *Alkalibacterium*) that were more abundant in feces collected in the cave (Supporting Information Figure S1).

## 4 DISCUSSION

Microbial community studies in bats are sometimes difficult to undertake because of the technical challenge in obtaining biological samples directly from bat individuals and ethical considerations. Here, we examined differences in bacterial communities inferred from direct and indirect sampling of bat's secretions (urine and feces). Results showed that even if sampling approach influenced the microbiota composition, niche specialization among excreta was well assessed by both methods, with especially high diversity in urine. This pattern has been previously evidenced in different insectivorous bat species in South Africa (Dietrich et al., 2017), and our results on the frugivorous bat *R. aegyptiacus* therefore suggest that niche specialization of microbiota is a general pattern in bats.

One striking difference between sampling approaches was the lower alpha‐diversity found with indirect sampling. A possible explanation is the potential degradation of material collected inside the cave, especially for feces droppings, as we cannot exactly tell the time since defection for these samples (even if we tried to minimize it by collecting the freshest droppings). A recent study showed indeed that a rapid reduction in fecal microbiota diversity can be expected after 1 hr post‐defecation, especially for anaerobic bacteria (Fofanov et al., 2018). For our indirect urine samples, material degradation should be limited as these samples were collected rapidly (within 20 min). However, shifts in microbiota composition seems to be time‐dependent, and long‐term decaying process of guano may in fact lead to increased bacterial diversity, because of the enrichment of bacteria involved in the recycling of the organic and inorganic matters (Banskar, Bhute, et al., 2016).

Therefore, we rather think that increased diversity in our direct samples was mainly the result of cross‐contamination between urine and fecal material from bats. Indeed, when bats urinate or defecate during handling, it is not always easy to isolate urine from feces and vice versa, especially for fruit bats (compared to the insectivorous one who make compact fecal pellets). We can therefore not exclude that the uro‐anal region was cross‐contaminated, and given the niche specialization, the mix of urine and fecal samples should increase the microbiota diversity of urine and feces samples directly collected from bats. This was supported by the higher percentage of overlapping phylotypes between urine and feces when direct sampling was used, and the enrichment of *Streptococcus* in urine (the most abundant phylotype in feces) with direct sampling. Finally, we cannot exclude that contamination from the skin and fur of bats may have occurred when collecting urine and feces. Indeed, the microbiota of bat's skin and fur is rich (Avena et al., 2016; Winter et al., 2017) and control samples (by swabbing the skin and fur of bats) should therefore be included in future investigations.

Based on beta‐diversity measurements, we found a structure in microbiota composition between sampling approaches, even if it was not significant for feces. The absence of significant results for feces may be explained by the limited number of feces samples collected directly (*n* = 5) and thus a lack of statistical power. Indeed, we observed that *R. aegyptiacus* do not defecate easily at the time of emergence, probably because they did not feed yet, and we were not able to obtain more fecal samples from bats. Difference in microbiota composition was illustrated by the enrichment of environmental bacteria in samples from the cave, such as *Gracilimonas*, *Salinisphera, Nesterenkonia, *and *Alkalibacterium*. This suggests that indirect sampling may favor contamination with bacteria present inside the cave. To limit this potential bias, the use of collection plates should be preferred, and for feces, we suggest that sampling at dawn should be more appropriate, as bats have fed and would give more fecal samples. The addition of negative controls (cave floor and surfaces) should also be included in the sampling scheme to further identify potential contamination using bioinformatic tools.

Finally, differences of the microbiota between sampling approaches may also result from a normal variation of the microbiota related to the phenology of bats. Indeed, direct and indirect samples were not collected at the exact same time of the day; direct samples were collected at dusk/night, while indirect samples were collected in the middle of the following day. As shown for the intestinal microbiota, in both mice and humans, diurnal oscillations (that are influenced by feeding rhythms) occur and lead to time‐specific compositional and functional profiles of the microbiota over the course of a day (Thaiss et al., 2014). Comparison of microbiota from indirect samples collected at different times of the day would allow to test if diurnal oscillations of microbiota also occur in bats.

In conclusion, we found that direct and indirect samplings of bats both captured the niche specialization pattern of bat's microbiota, despite some differences in alpha‐ and beta‐diversity. Our comparative study allowed us to suggest potential contamination biases in both approaches, and to propose enhanced sampling protocols for future investigations of bat's microbial community dynamics.

## CONFLICT OF INTEREST

None declared.

## AUTHOR CONTRIBUTION

M.D. and W.M. conceived and designed the study, and participated in field sampling. M.D. performed processing of samples in the lab and analysis of data. M.D. drafted the manuscript. Both authors edited the manuscript and gave their approval for publication.

## Data Availability

The Illumina sequencing reads and the supplemental figures and tables are publicly available for download on Dryad https://doi.org/10.5061/dryad.k2v2006.
