# Supplementary material for: Studying the microbiota of bats: Accuracy of direct and indirect samplings
Source: Ecol Evol. 2019 Jan 24;9(4):1730–5. doi: 10.1002/ece3.4842 (PMC6392341; doi:10.1002/ece3.4842)
Supplement: Supplementary file 1 [file ECE3-9-1730-s001.pdf]

Supplementary Material

Studying the microbiota of bats: accuracy of direct and indirect samplings

Muriel Dietrich & Wanda Markotter

ECOLOGY & EVOLUTION

Figure S1. Results of LefSe analysis for urine (left) and faeces (right) samples. Phylotypes enriched in direct and indirect samplings are colored by green and red, respectively. Only phylotypes meeting a LDA significant threshold > 2 and p > 0.05 are shown.

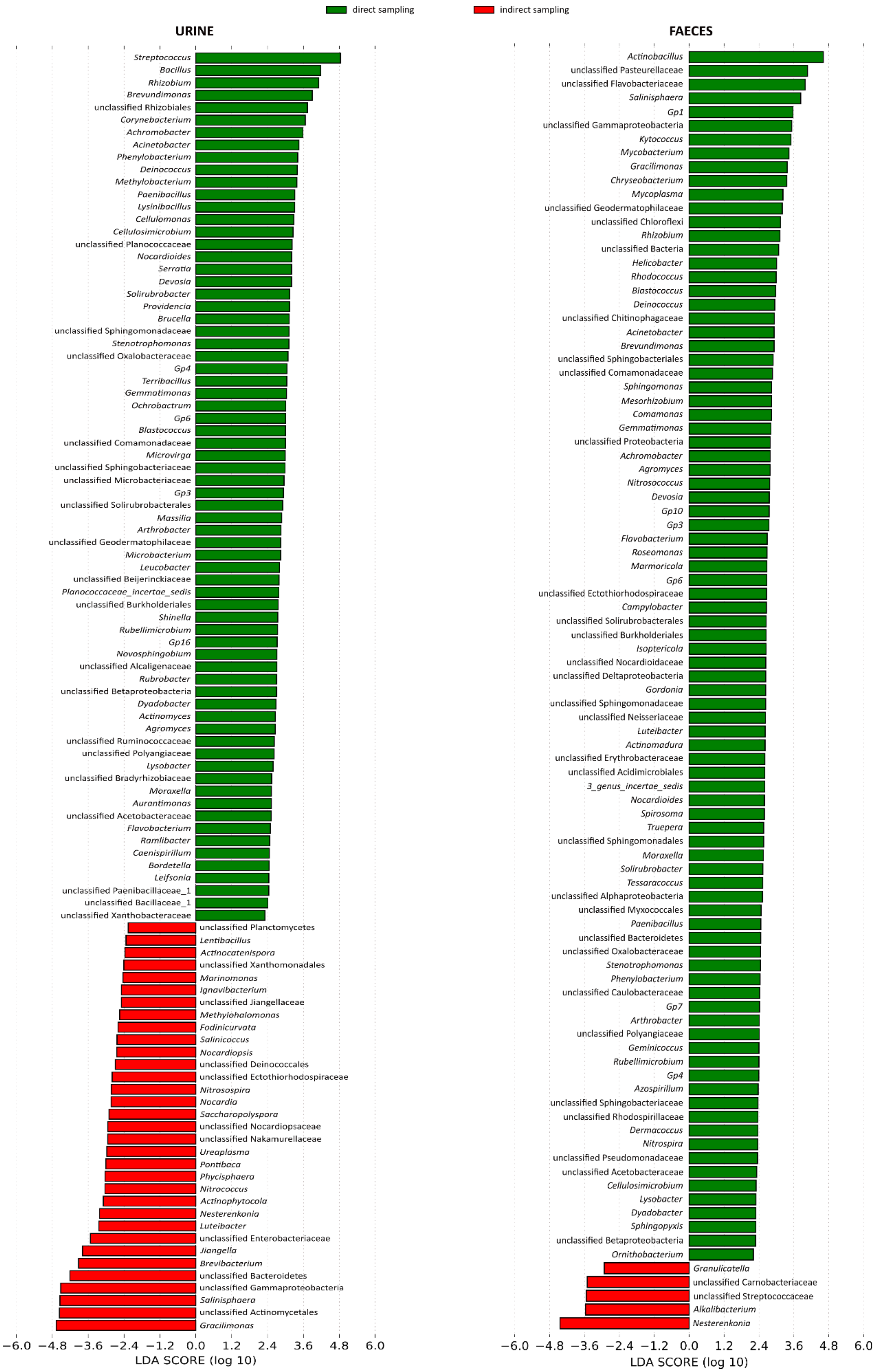
direct sampling
indirect sampling
URINE
FAECES
LDA SCORE (log 10)
LDA SCORE (log 10)
