# Supplementary material for: Studying the microbiota of bats: Accuracy of direct and indirect samplings
Source: Ecol Evol. 2019 Jan 24;9(4):1730–5. doi: 10.1002/ece3.4842 (PMC6392341; doi:10.1002/ece3.4842)
Supplement: Supplementary file 2 [file ECE3-9-1730-s002.docx]

**Supplementary Material**

**Studying the microbiota of bats: accuracy of direct and indirect samplings**

Muriel Dietrich & Wanda Markotter

**ECOLOGY & EVOLUTION**

**Table S1. Description of GLMs used for the analysis of alpha-diversity.** N gives the number of samples included in the analysis. Excreta refers to the sample type (faeces/urine). "Full model" gives the complete set of explanatory variables tested (and their interactions) included in the model. "Minimal model" gives the model containing only the significant variables and their interactions. All starting GLMs were simplified by backward stepwise elimination of non-significant terms (*p* > 0.05), to obtain the minimum adequate model. The χ² significant values given in the results are those obtained when the variable is deleted from the minimal model.

| **Analysis** | **Model nb.** | **N** | **Full model** | **Minimal model** |
| --- | --- | --- | --- | --- |
| Alpha-diversity between excreta samples | GLM_1_ | 35 | Excreta*Session | Excreta |
| Alpha-diversity between sampling approaches in urine | GLM_2_ | 24 | Sampling*Session | Session |
| Alpha-diversity between sampling approaches in faeces | GLM_3_ | 11 | Sampling*Session | null |
